# Cerebellar volumes’ selective association with MoCA over MMSE: a diagnostic insight into mild cognitive impairment and mild dementia

**DOI:** 10.1007/s10072-026-08816-9

**Published:** 2026-01-21

**Authors:** Maria Devita, Chiara Ceolin, Chiara Begliomini, Michela Sarlo, Alessandra Coin, Alessandra Bertoldo, Giuseppe Sergi, Daniela Mapelli, Marina De Rui

**Affiliations:** 1 Department of General Psychology (DPG), University of Padua, Via Venezia 8, Padua, Italy; 2 Geriatrics Unit, Department of Medicine (DIMED), University of Padua, Via Giustiniani 2, Padua, Italy; 3 Department of Communication Sciences, Humanities and International Studies, University of Urbino Carlo Bo, Via Saffi 15, 61029 Urbino, Italy; 4 Department of Information Engineering, University of Padua, Via Gardenigo 6/B, Padua, Italy; 5 Padova Neuroscience Center, University of Padua, Via Orus 2/B, Padua, Italy

**Keywords:** Cerebellum, Mild cognitive impairment, Cognitive cerebellum, MoCA

## Abstract

**Introduction:**

Linked to motor control, cerebellum is increasingly recognized for its role in cognition and neurodegenerative disorders.

**Methods:**

This retrospective study investigates associations between cerebellar volumes and cognitive screening tools—the Mini-Mental State Examination (MMSE) and the Montreal Cognitive Assessment (MoCA)—in individuals with mild cognitive impairment (MCI) or mild dementia.

**Results:**

MoCA scores showed significant positive correlations with cognitive-related cerebellar regions, particularly the left Crus I lobule (r = 0.40, *p* = 0.02) and total Crus I volume (r = 0.36, *p* = 0.04). Regression analysis confirmed associations with the left Crus I (β = 0.08, *p* = 0.02) and right VIIB lobule (β = 0.033, *p* = 0.032), while MMSE scores correlated only with right Lobule X thickness (r = -0.35, *p* = 0.04).

**Discussion:**

These findings suggest MoCA may better detect cerebellar-related cognitive impairments, underscoring the importance of including cerebellar evaluation in the early diagnosis of dementia.

## Introduction

For decades, the role of cerebellum has been completely neglected in neurodegenerative disorders.

As a matter of fact, the still enigmatic hypothesis of its involvement in dementia has faced numerous challenges, and several plausible explanations may account for the scarcity of cerebellar findings in the dementia literature. As already synthesized in Devita and colleagues [1], the cerebellum's complex structure and caudal location pose significant technical issues for imaging modalities, often compromising the acquisition of high-quality images [2]. Secondly, the prevailing assumption that the cerebellum is spared from neurodegeneration has led many studies to exclude it from their analyses [3]. Furthermore, due to this same assumption, PET studies frequently utilize the cerebellum as a reference region (see [4]), which may have resulted in underestimation of dementia-related cerebellar deterioration. Nevertheless, research keeps leading to the intriguing hypothesis that the cerebellum not only plays a role in neurodegenerative diseases, but also that it may be involved in their early phases (for a review, see [1]). What is more, preliminary evidence suggests that structural alterations of the cerebellum might potentially have some prognostic value as well; similarly, cerebellar atrophy can be already present in MCI or asymptomatic patients, but in certain cases it could also be a predictor of conversion to major neurocognitive impairment [5]. Yet, clinicians frequently rely on diagnostic tools like the Mini-Mental State Examination (MMSE [6]), which has notable limitations in detecting subtle and early cognitive impairments, particularly in executive functions and visuospatial abilities [7]. This lack of sensitivity may lead to incorrect diagnoses and overlook specific cerebellar-related cognitive deficits, such as those associated with the Cerebellar Cognitive Affective Syndrome (CCAS), characterized by impairments in executive function, linguistic processing, spatial cognition, and affect regulation [8, 9]. As a result, the oversight of cerebellar involvement in neurodegenerative disorders may impede early diagnosis and treatment, underscoring the need for further investigation into this organ. Enhanced diagnostic tools and a greater focus on cerebellar cognitive impairments may be thus crucial for accurate diagnosis and early management of the disease. Currently, the MMSE and the Montreal Cognitive Assessment (MoCA [10]) tests are the most administered screening tools for cognitive impairment [11, 12]. Although previous studies have identified associations between both MMSE and MoCA and cerebellar volumes, to the best of our knowledge, no literature exists that directly compares these two measures in relation to cerebellar volumes. Therefore, this study aims to investigate the comparative associations between MMSE and MoCA scores with cerebellar volumes, in order to elucidate which cognitive assessment tool exhibits a stronger correlation with neuroanatomical data. This will highlight the significance of cerebellar involvement in mild cognitive impairment and mild dementia.

## Materials and methods

For this retrospective observational study, 33 participants were recruited from the outpatient clinic Center for Cognitive Decline and Dementia (CDCD), Padua Hospital, Department of Medicine. Inclusion criteria were: diagnosis of Mild Cognitive Impairment (MCI); mild or mild-moderate dementia i.e. Alzheimer’s disease like or mixed dementia (last MMSE score between 18 and 24 inclusive), according with the recommendations of the American Psychiatry Association's Diagnostic and Statistical Manual of Mental Disorders 5th Edition (DSM-V); patients’ informed consent to participate to the study (even, if necessary, in the presence of a caregiver or appointed legal guardian—e.g., legal administrator if applicable-); psycho-physical ability to perform the required tasks. Exclusion criteria were: diagnosis of advanced cognitive decline; severe sensory deficits; presence of psychiatric disorders; poor quality of neuroimaging images.

Sociodemographic characteristics were obtained retrospectively by consulting medical records from the CDCD. All patients underwent clinical, cognitive and neuroimaging evaluation; MRI acquisitions were conducted within three months following the cognitive assessment (this interval corresponds to the typical operational scheduling of the Neuro-Radiology Unit, which, in addition to research commitments, must accommodate clinical and routine diagnostic activities).

### Neuropsychological assessment

To the aims of this study, both the MoCA and the MMSE screening test were administered and analysed to compare the association between these commonly used tools and cerebellar volumes.

- The Montreal Cognitive Assessment (MoCA [10]) is a rapid screening tool validated for Mild Cognitive Impairment, consisting of 30 questions exploring various cognitive domains. The cognitive domains explored include attention, concentration, executive functions, memory, language, visuo-constructive skills, abstraction, calculation, and orientation.
- The Mini Mental State Examination (MMSE [6]) consists of 30 questions covering 7 cognitive areas (orientation in space and time, word registration, attention and calculation, recall, language, constructive praxis).

### MR images acquisition and preprocessing

Structural images of the cerebellum were acquired using magnetic resonance imaging (MRI) on a 3.0 T scanner (Ingenia, Philips Medical Systems, Best, Netherlands) equipped with a 32-channel head coil. High-resolution T1-weighted (T1w) images were obtained with a 3D-TFE sequence with compressed sensing (factor 3.5), adopting the following parameters: Time of Repetition (TR) = 6.7 ms, Time of Echo (TE) = 3.0 ms, flip angle = 8°, Field Of View (FOV) = 240 × 240 mm^2^, and an isotropic resolution of 1 mm. The preprocessing of T1w images involved multiple steps to ensure accurate cerebellar parcellation. First, N4 bias field correction [13] was applied using the Advanced Normalization Tools [14]. Cerebellum and brainstem masks were generated from the resulting images and used to isolate the relevant anatomical structures. Any residual spurious voxels from masking were removed with a custom Matlab script [15]. To further refine cerebellar isolation, the Spatially Unbiased Infra-Tentorial (SUIT [16]) Matlab toolbox was employed to define a bounding box, which was applied to the masked-T1w image to extract a cropped image of the cerebellar volume. This volume was then registered to the SUIT template built according to the Montréal Neurological Institute (MNI) stereotaxic space through a combination of affine and non-linear transformations using ANTs. Subsequently, the inverse transformation was applied to the SUIT anatomical cerebellar parcellation, bringing it into the subject’s native T1w space. To ensure accurate delineation of cerebellar structures, parcellations were visually inspected by an expert neuroradiologist using FSLeyes [17] and manually corrected if necessary by overlaying cerebellar lobule parcellations onto the structural T1w image. Since the SUIT toolbox performs the segmentation including both gray and white matter, an additional step was performed to exclude white matter and focus exclusively on gray matter. Individual white matter masks were generated using FreeSurfer 7.1 [18] (https://surfer.nmr.mgh.harvard.edu/), and brain extraction with ANTs was performed on the T1w image to estimate the affine transformation aligning it with the FreeSurfer-derived brain image. This transformation was then applied to the cerebellar parcellation to refine tissue classification. The white matter mask was subsequently used to isolate gray matter regions within the cerebellar parcellation. Finally, cerebellar lobule volumes were computed using a customized in-home Matlab script and are reported in Table 1.

**Table 1:** Patient characteristics, cerebral and cerebellar volumes

		Sex		
Variable	Overall, *N* = 33^1^	Males	Females	*p*-value
AGE [years]	77.2 (3.7)	78.2 (3.7)	76.7 (3.6)	0.50
EDUCATION [years]	7.3 (3.0)	8.1 (3.1)	6.9 (3.0)	0.33
MMSE	24.6 (4.1)	27.0 (2.6)	23.6 (4.3)	0.041
MOCA	19.6 (6.0)	23.1 (4.7)	18.2 (6.0)	0.054
*Cerebral volumes*				
TIV	1,332 (138)	1,453 (110)	1,280 (115)	< 0.001
GM	526 (49)	550 (62)	515 (40)	0.075
WM	437 (54)	497 (38)	412 (36)	< 0.001
CSF	369 (76)	405 (69)	353 (74)	0.10
*Cerebellar volumes*				
Cerebellum total volume [cm^3^]	107 (10)	115 (13)	104 (7)	0.042
White Matter total volume [cm^3^]	37.5 (3.9)	41.1 (4.4)	36.0 (2.4)	0.002
Grey Matter total volume [cm^3^]	70 (7)	74 (9)	68 (5)	0.10
Lobules I-II total volume [cm^3^]	0.06 (0.03)	0.07 (0.04)	0.05 (0.03)	0.063
Lobule III total volume [cm^3^]	0.76 (0.14)	0.83 (0.18)	0.73 (0.11)	0.22
Lobule IV total volume [cm^3^]	2.51 (0.30)	2.64 (0.31)	2.46 (0.29)	0.14
Lobule V total volume [cm^3^]	4.45 (0.59)	4.86 (0.66)	4.28 (0.47)	0.012
Lobule VI total volume [cm^3^]	11.18 (1.71)	11.71 (2.34)	10.94 (1.35)	0.34
Crus I total volume [cm^3^]	15.98 (2.25)	16.84 (2.53)	15.60 (2.07)	0.13
Crus II total volume [cm^3^]	10.59 (1.72)	11.24 (1.80)	10.30 (1.64)	0.19
Lobule VIIB total volume [cm^3^]	6.54 (0.86)	7.16 (0.99)	6.27 (0.65)	0.016
Lobule VIIIA total volume [cm^3^]	7.43 (0.96)	7.69 (1.17)	7.31 (0.85)	0.34
Lobule VIIIB total volume [cm^3^]	4.91 (0.61)	5.07 (0.67)	4.84 (0.59)	0.32
Lobule IX total volume [cm^3^]	4.17 (0.61)	4.47 (0.74)	4.05 (0.52)	0.068
Lobule X total volume [cm^3^]	0.96 (0.13)	1.04 (0.10)	0.93 (0.12)	0.012

^1^Mean (SD) or Frequency (%)

## Statistical analyses

Cerebellum was divided into “cognitive” (Crus I and II, lobule VII, the adjacent lobule VI and IX), and “motor” (I-V, VIII lobules, adjacent lobule VI) according to the previous publication [1]. Participants’ characteristics were summarized using means ± standard deviations for normally distributed quantitative variables and medians for variables with non-normal distributions. Normality was assessed using the Shapiro–Wilk test. Categorical variables were presented as frequencies and percentages. Comparative analyses between groups were conducted using Student's t-test and Mann–Whitney test for continuous variables, and Chi-square test and Fisher's exact test for categorical variables. Multiple linear regression, employing a stepwise forward procedure, was utilized to explore the relationship between cerebellar volumes and variables of interest. Statistical significance was considered at *p* < 0.05. Analyses were conducted using SPSS software (version 29) and R version 4.1.1 [19]. All linear regression analyses were adjusted for age, sex, and total intracranial volume (TIV). Model assumptions, including normality of residuals (Shapiro–Wilk test), linearity, homoscedasticity, and multicollinearity (VIF < 5), were verified and satisfied prior to interpretation, confirming the adequacy of the linear modeling approach.

## Results

Table 1 presents the characteristics of the sample, stratified by sex.

Men had significantly lower MMSE scores than women (27.0 ± 2.6 vs. 23.6 ± 4.3, *p* = 0.04). Regarding cerebral volumes, women exhibited lower values for total intracranial volume (TIV), white matter volume, and the right opercular part of the inferior frontal gyrus (OpIFG) (see Supplementary Table 1). For cerebellar volumes, men had greater total cerebellar volume and white matter volume. Sex differences were particularly observed in specific lobules, including Lobule V (total volume: 4.86 ± 0.66 vs. 4.28 ± 0.47, *p* = 0.012), VIIB (total volume: 7.16 ± 0.99 vs. 6.27 ± 0.65, *p* = 0.016), and X (total volume: 1.04 ± 0.10 vs. 0.93 ± 0.12, *p* = 0.012). Additionally, differences in thickness were recorded for the right Crus I and Crus II, as well as for lobule VIIB bilaterally (see Supplementary Table 1).

**Fig. 1:**
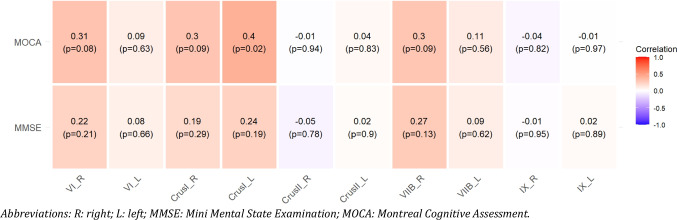
Heatmap correlation between MMSE and MOCA questionnaires, and cognitive cerebellar volumes. Abbreviations: R: right; L: left; MMSE: Mini Mental State Examination; MOCA: Montreal Cognitive Assessment

To explore potential associations between MOCA and MMSE scores and both cerebral and cerebellar volumes, correlation analyses were performed. A significant positive correlation was found between MOCA scores and the left Crus I lobule (r = 0.40, *p* = 0.02) and total Crus I volume (r = 0.36, *p* = 0.04). Additionally, MOCA scores showed a correlation with right VI (r = 0.31, *p* = 0.08), right Crus I (r = 0.3, *p* = 0.09), and right VIIB (r = 0.3, *p* = 0.09) lobules, although these associations did not reach statistical significance (Fig. 1).

**Fig. 2:**
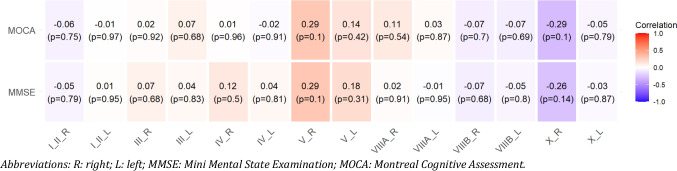
Heatmap correlation between MMSE and MOCA questionnaires, and motor cerebellar volumes. Abbreviations: R: right; L: left; MMSE: Mini Mental State Examination; MOCA: Montreal Cognitive Assessment

No significant correlations were found between motor cerebellar volumes and MOCA or MMSE scores (Fig. 2). MMSE scores were significantly correlated only with right Lobule X thickness (r = −0.35, p = 0.04). Regarding cerebral volumes, no significant correlations were observed for either MMSE or MOCA scores (Table 2).

**Table 2:** Correlation coefficients between MMSE and MOCA questionnaires, and cerebral volumes

	MMSE		MOCA	
	r	*p*	r	*p*
TIV	0,13	0,48	0,22	0,22
GM	0,07	0,66	0,19	0,29
WM	0,26	0,13	0,27	0,12
CSF	−0,01	0,96	0,07	0,67
Right Caudate	0,17	0,32	0,21	0,23
Left Caudate	0,05	0,77	0,07	0,67
Right Pallidum	−0,03	0,84	−0,01	0,97
Left Pallidum	−0,18	0,31	−0,16	0,37
Right Putamen	0,11	0,53	0,07	0,69
Left Putamen	0,15	0,38	0,06	0,72
Right Hippo	0,26	0,13	0,23	0,18
Left Hippo	0,18	0,29	0,17	0,33
Right PHG	0,22	0,21	0,24	0,17
Left PHG	0,17	0,34	0,15	0,37
Right entorhinal	0,23	0,18	0,27	0,12
Left entorhinal	0,07	0,68	0,09	0,59
Right STG	0,03	0,84	−0,04	0,8
Left STG	−0,14	0,41	−0,15	0,37
Right MFC	−0,04	0,81	−0,02	0,91
Left MFC	−0,1	0,56	−0,11	0,52
Right MOrG	0,14	0,41	0,1	0,56
Left MOrG	0,09	0,59	0,04	0,82
Right SFG	0,16	0,37	0,1	0,54
Left SFG	−0,08	0,64	−0,07	0,68
Right MSFG	0,16	0,36	0,16	0,35
Left MSFG	0,12	0,49	0,16	0,36
Right MFG	0,1	0,57	0,02	0,91
Left MFG	0,03	0,85	0	0,96
Right OpIFG	0,12	0,5	0,15	0,39
Left OpIFG	−0,19	0,28	−0,21	0,24
Right OrIFG	−0,06	0,73	−0,12	0,49
Left OrIFG	0,11	0,5	0,03	0,84
Right TrIFG	0,12	0,49	0,03	0,83
Left TrIFG	0,01	0,95	−0,05	0,74
Right PCu	0,07	0,69	0,11	0,52
Left PCu	0,05	0,75	0,05	0,75
Right AnG	0,05	0,74	−0,01	0,95
Left AnG	−0,08	0,63	−0,1	0,55

Abbreviations: *p p*-value, *TIV* total intracranial volume, *GM* grey matter, *WM* white matter, *CSF* cerebrospinal fluid, *Right Caudate* right caudate nucleus, *Left Caudate* left caudate nucleus, *Right Pallidum* right pallidum, *Left Pallidum* left pallidum, *Right Putamen* right putamen, *Left Putamen* left putamen, *Right Hippo* right hippocampus, *Left Hippo* left hippocampus, *Right PHG* right parahippocampal gyrus, *Left PHG* left parahippocampal gyrus, *Right entorhinal* right entorhinal cortex, *Left entorhinal* left entorhinal cortex, *Right STG* right superior temporal gyrus, *Left STG* left superior temporal gyrus, *Right MFC* right medial frontal cortex, *Left MFC* left medial frontal cortex, *Right MOrG* right medial orbitofrontal gyrus, *Left MOrG* left medial orbitofrontal gyrus, *Right SFG* right superior frontal gyrus, *Left SFG* left superior frontal gyrus, *Right MSFG* right medial superior frontal gyrus, *Left MSFG* left medial superior frontal gyrus, *Right MFG* right middle frontal gyrus, *Left MFG* left middle frontal gyrus, *Right OpIFG* right opercular inferior frontal gyrus, *Left OpIFG* left opercular inferior frontal gyrus, *Right OrIFG* right orbitofrontal inferior frontal gyrus, *Left OrIFG* left orbitofrontal inferior frontal gyrus, *Right TrIFG* right triangular inferior frontal gyrus, *Left TrIFG* left triangular inferior frontal gyrus, *Right PCu* right precuneus, *Left PCu* left precuneus, *Right AnG* right angular gyrus, *Left AnG* left angular gyrus

Finally, a linear regression analysis adjusted for age and sex was conducted to investigate potential associations between MOCA scores and cerebellar volumes (Table 3).

**Table 3:** Linear regression analysis investigating association between MOCA scores and cerebellar volumes

Volumes	Intercept (β, *p*)	MOCA (β, *p*)	R^2^ (Adj. R^2^)
Lobule V Right	1.95 (*p* < 0.001)	0.015 (*p* = 0.105)	0.08 (0.051)
Lobule VI Right	4.60 (*p* < 0.001)	0.049 (*p* = 0.076)	0.10 (0.072)
Crus I Right	6.73 (*p* < 0.001)	0.059 (*p* = 0.088)	0.091 (0.062)
Crus I Left	6.52 (*p* < 0.001)	0.080 (*p* = *0.020*)	0.163 (0.136)
Lobule VII B Right	2.48 (*p* < 0.001)	0.033 (*p* = *0.032*)	0.158 (0.102)
Lobule X Right	0.541 (*p* < 0.001)	−0.003 (*p* = 0.102)	0.084 (0.055)

All models were adjusted for age, sex, and TIV. Diagnostic checks confirmed the validity of model assumptions, supporting the use of linear regression

Using a stepwise approach, MOCA scores were significantly associated with cognitive-related cerebellar regions, specifically the left Crus I lobule (β = 0.08, *p* = 0.02) and the right VIIB lobule (β = 0.033, *p* = 0.032). Additionally, a trend towards significance was observed with the right Lobule VI (β = 0.049, *p* = 0.076) and the right Crus I lobule (β = 0.059, *p* = 0.088). In contrast, no significant associations were found for MMSE scores (see Supplementary Table 1).

## Discussion

The present study aimed to compare the associations between Mini-Mental State Examination (MMSE) and Montreal Cognitive Assessment (MoCA) scores with cerebellar volumes in individuals with mild cognitive impairment (MCI) or mild dementia. Our findings revealed a selective and statistically significant association between MoCA scores and cerebellar regions implicated in higher cognitive functions—specifically, the left Crus I and right Lobule VIIB. In contrast, MMSE scores were weakly associated only with the thickness of right Lobule X, a region with limited established cognitive roles. These results suggest that MoCA, but not MMSE, may be sensitive enough to capture cerebellar-related cognitive changes in the early phases of neurodegenerative conditions. This is consistent with prior studies indicating that the cerebellum, traditionally viewed as a motor structure, plays a crucial role in cognition through its extensive connections with the cerebral cortex. Anatomically and functionally, the cerebellum forms reciprocal circuits with several key associative brain areas via the cerebro-cerebellar loops, particularly through the dentate nucleus, thalamus, and prefrontal, parietal, and temporal cortices [20–22]. Notably, Crus I and II—regions found to correlate with MoCA in our study—are especially connected with the dorsolateral prefrontal cortex (DLPFC) and the posterior parietal cortex, areas implicated in executive function, working memory, and visuospatial attention [9, 23]. The right Lobule VIIB, also associated with MoCA in our analysis, is part of the cerebellar network that interacts with language-related regions such as Broca’s area, contributing to inner speech and verbal fluency [24]. Through the cortico-ponto-cerebellar and cerebello-thalamo-cortical pathways, these lobules integrate information from associative cortices and modulate higher-order cognitive functions [25, 26]. Moreover, neuroimaging studies have consistently shown that cerebellar atrophy, particularly in Crus I/II and Lobule VI, is present in early Alzheimer's disease (AD) and MCI, even when classical cortical areas like the hippocampus remain relatively preserved [27, 28]. The cerebellum thus appears not only to be affected in neurodegenerative conditions but may exhibit pathological changes earlier than some cortical areas, thereby providing a potential early biomarker for disease progression. Interestingly, no significant associations were found between either MMSE or MoCA scores and cerebral volumes in our cohort. This lack of correlation with traditional cortical markers of dementia, such as the hippocampus or entorhinal cortex, could indicate that in early neurodegeneration, cerebellar alterations may precede detectable cerebral atrophy. These findings underscore the importance of considering extracortical structures in cognitive assessments, especially in the preclinical and early symptomatic stages of dementia. MoCA’s sensitivity to these cerebellar-cortical dynamics may stem from its broader assessment of executive functions, attention, abstraction, and visuospatial processing—domains where cerebellar contributions are increasingly recognized [29, 30]. Conversely, the MMSE primarily evaluates orientation, recall, and language fluency, offering limited insight into subtle deficits in executive or visuospatial processing, hence its weaker association with cerebellar regions. From a clinical standpoint, our findings highlight the potential of cerebellar volumetric assessment as an adjunct biomarker in the diagnostic and prognostic evaluation of cognitive impairment [31, 32]. In clinical practice, volumetric MRI protocols could be expanded to include cerebellar segmentation, allowing clinicians to detect early extracortical changes that might otherwise go unnoticed when focusing solely on hippocampal or cortical metrics [33]. Furthermore, the observed association between MoCA scores and specific cerebellar lobules suggests that MoCA—and potentially other cognitive screening tools emphasizing executive and visuospatial components—may provide a more accurate reflection of cerebellar-related cognitive decline [34, 35]. This could inform the choice of cognitive assessments in clinical settings, especially when dealing with atypical or non-amnestic forms of MCI. These results also open new perspectives for individualized rehabilitation strategies. Identifying patients with cerebellar involvement may allow targeted cognitive interventions focusing on executive, visuospatial, and linguistic domains—functions supported by the cerebellar circuitry [36]. Additionally, neuromodulation techniques such as transcranial magnetic stimulation (TMS) or transcranial direct current stimulation (tDCS) over cerebellar regions could be explored to enhance cognitive outcomes in early neurodegenerative stages [37, 38].

### Limitations

Our study has certainly some limitations. First of all, the sample size is small, which may have affected the power to detect significant correlations between MMSE scores and cerebellar volumes. Secondly, the absence of a control group represents another significant limitation, and therefore our results should be considered preliminary and interpreted with caution. Future studies should aim to replicate these findings in larger cohorts and explore the longitudinal impact of cerebellar volume changes on cognitive decline. Additionally, integrating advanced neuroimaging techniques with detailed neuropsychological assessments could provide deeper insights into the cerebellum's role in neurodegenerative diseases.

## Conclusions

In conclusion, our study highlights the significance of cerebellar involvement in cognitive impairments and suggests that the MoCA test is more sensitive to these changes than MMSE. These findings support the need for a more comprehensive approach to diagnosing and managing neurodegenerative disorders, including a focus on cerebellar cognitive functions. By integrating cerebellar assessments into clinical practice, clinicians may improve diagnostic accuracy and develop more targeted therapeutic strategies for patients with mild cognitive impairment and early dementia.

## Data Availability

Data will be available by the authors on reasonable request.
